# Exploring the factors contributing to increase in facility child births in Bangladesh between 2004 and 2017–2018

**DOI:** 10.1016/j.heliyon.2023.e15875

**Published:** 2023-05-02

**Authors:** Md Sohel Rana, Sk Masum Billah, Mohammed Moinuddin, Md Abu Bakkar Siddique, Md Mobarak Hossain Khan

**Affiliations:** a Department of Statistics, Comilla University, Kotbari, 3506, Cumilla, Bangladesh; b Maternal and Child Health Division, International Centre for Diarrhoeal Disease Research, Bangladesh (icddr'b), Dhaka, Bangladesh; c School of Public Health, The University of Sydney, Australia; d Faculty of Health and Social Care, Edge Hill University, Lancashire, UK; e Department of Social Relations, East West University, Aftabnagar, Dhaka, Bangladesh

**Keywords:** Skilled birth attendants, Classical decomposition, Facility child birth, Predicted change, Bangladesh

## Abstract

**Background:**

Although Bangladesh has gained rapid improvement in births at health facilities, yet far behind to achieve the SDG target. Assessing the contribution of factors in increased use of delivery at facilities are important to demonstrate.

**Objective:**

To explore the determinants and their contribution in explaining increased use of facility child births in Bangladesh.

**Participants:**

Reproductive-aged women (15–49 years) of Bangladesh.

**Methods and materials:**

We used the latest five rounds (2004, 2007, 2011, 2014, 2017–2018) of Bangladesh Demographic and Health Surveys (BDHSs). The regression based classical decomposition approach has been used to explore the determinants and their contribution in explaining the increased use of facility child birth.

**Results:**

A sample of 26,686 reproductive-aged women were included in the analysis, 32.90% (8780) from the urban and 67.10% (17,906) from the rural area. We observed a 2.4-fold increase in delivery at facilities from 2004 to 2017–2018, in rural areas it is more than three times higher than the urban areas. The change in mean delivery at facilities is about 1.8 whereas, the predicted change is 1.4. In our full sample model antenatal care visits contribute the largest predicted change of 22.3%, wealth and education contributes 17.3% and 15.3% respectively. For the rural area health indicator (prenatal doctor visit) is the largest drivers contributing 42.7% of the predicted change, hereafter education, demography and wealth. However, in urban area education and health contributed equally 32.0% of the change followed by demography (26.3%) and wealth (9.7%). Demographic variables (maternal BMI, birth order, age at marriage) contributing more than two-thirds (41.2%) of the predicted change in the model without the health variables. All models showed more than 60.0% predictive power.

**Conclusion:**

Health sector interventions should focus both coverage and quality of maternal health care services to sustain steady improvements in child birth facilities.

Introduction

### 1 Background

Increase in facility child birth usually measured by increased in the use of delivery assisted by skilled birth attendants. Regardless of precipitous escalation of facility child birth, maternal and child mortality and morbidity are hitherto unsatisfactorily high in many countries. We observed 44% global maternal deaths reduction from 1990 to 2015 [1]. Around 73% of maternal death occurred due to the obstetric complications [2]. Therefore, key intervention to prevent these proliferate number of maternal death is considered as the universal access of skilled birth attendants and emergency obstetric care [3,4]. The risk of maternal and neonatal deaths can be reduced by ensuring the delivery by skilled birth attendants (SBAs) [[4], [5], [6], [7], [8], [9], [10], [11]]. So, for achieving sustainable development goals (SDGs), it is undoubtedly essential to implement the universal coverage of skilled delivery care services [[12], [13], [14]]. Birth attended by skilled attendants (SBAs) is considered as the “most important determinants in preventing maternal and neonatal deaths” [15]. For designing and implementing equitable interventions in health and healthcare the identification of determinants is inevitable [16] .

Although Bangladesh is a low-income country and its economy is emerging only for the last few years, it has gained notable achievement in the public health sectors for the last couple of decades [17,18]. Bangladesh showed outstanding performance in reduction of maternal and child mortality to meet the target of Millennium Development Goals (MDGs) in 2015. For instance, in 1990 the maternal mortality ratio and infant mortality ratio were about 507 and 149 per 100,000 live births respectively. Those rates have declined to 209 and 53 respectively in 2010 [19]. That is why it is believe to be that Bangladesh is in the track to reach the Sustainable Development Goals (SDGs) [[20], [21], [22]]. Bangladesh National Strategy for Maternal Health 2014–2024 make the target to reduce the existing MMR of 176 per 100,000 live births to 50 by 2024 [23,24]. However, skilled birth attendants (SBAs) services performance in delivery is poor in Bangladesh which is 42.1%, still quite low, according to the global standard, and needs to be a minimum of 50% [25]. Traditionally most of the deliveries take place at home; yet 48.2% of total births occurred at household level and with the help of traditional birth attendants (TDAs). Birth attended by doctors are less than one third of total birth [25]. Thus a majority of women do not get the service of skilled birth facilities and completely rely on traditional birth attendants (TBAs). These are ultimate threats and influential reasons to maternal health. Bangladesh as a densely populated country, estimations based on the current fashion have revealed that every year about 124,392 women are at birth related mortality risk. Most of the maternal and neonatal deaths account for delivery assisted by unskilled birth attendants and relatives with no professional training [25,26]. It is reported that births occurred at home are unsafe, unhygienic and less prepared for essential new born care relative to the births at health facilities [22].

Wealth status of the mother as well as the family have significant influence on facility based deliveries. Multiple studies revealed that rich women or women from rich family are more likely to have birth delivered at a health facility compared to women with poor wealth condition [[27], [28], [29]]. Education taken into consideration as a determinant, it is documented that uneducated or less educated women compared to the women having higher levels of education, are less likely to understand the importance and utilise facility based delivery care services [30].

The objective of this study is to determine the factors associated to the increase of facility live births in Bangladesh and exploring their comparative importance by estimating the contribution of the factors in explaining increase in delivery assisted by SBAs using the latest five rounds of BDHS. A limited number of studies assessed the significant determinants of delivery assisted by skilled birth attendants in Bangladesh where a few did decomposition based on concentration index (CI) [[31], [32], [33]], but to our knowledge no studies used the regression based classical decomposition analysis technique to explore importance of specific factors in explaining the increasing trend among the women to utilise the aforementioned services. By determining the contribution of each factors in increased use of facility child birth we can address interventions to improve sectors those are considered less. Moreover, as policy implications we might shed light importantly on those factors which are the most influential drivers for improvement in the utilization of skilled birth attendants in delivery.

## 2 Methods

### 2.1 Study design and data sources

**Fig. 1:**
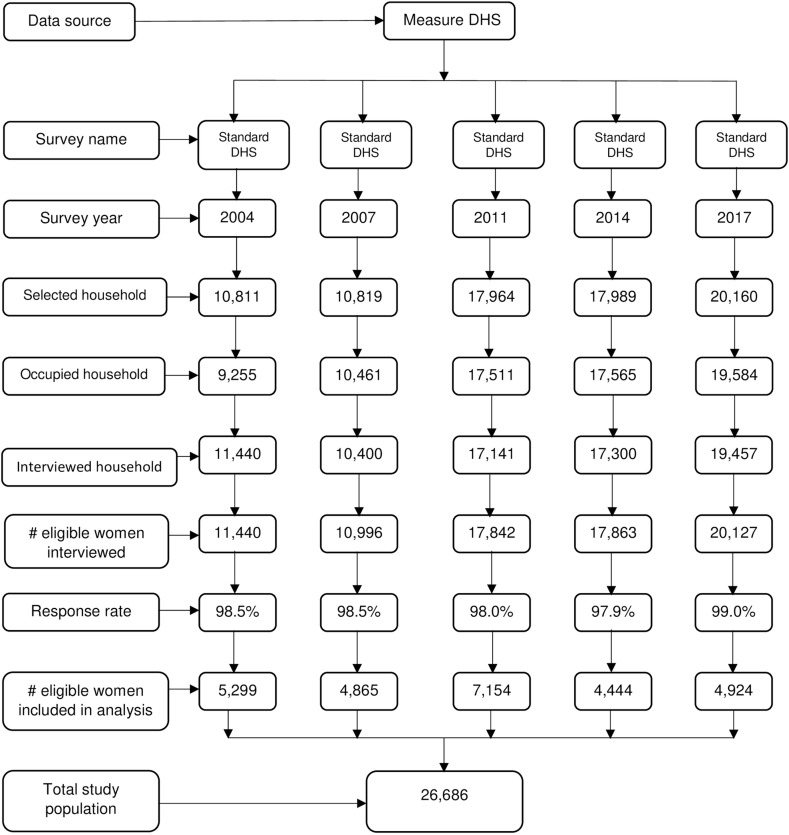
Distribution of study sample (N = 26,686).

We used data from the latest five rounds of Bangladesh Demographic and Health Surveys (BDHSs) occurred over a time span of 14 years in between 2004 and 2017–2018. The BDHS is a nationally representative and cross-sectional household survey, which is a part of Demographic and Health Survey (DHS) program and were conducted approximately every three years in all eight administrative regions of Bangladesh. The details of sampling procedures can be found elsewhere [[34], [35], [36], [37], [38]]. We pooled data from the surveys which resulted in an analytic sample of 26,686 women (See Fig. 1) with at least one live birth. As the BDHSs data cover a broad range of the hypothesized drivers of change in child birth facility, these are well suited to fulfil our study objectives.

### 2.2 Variables

#### 2.2.1 Outcome variables

The outcome variable in this study is ‘delivery assisted by skilled birth attendants (SBAs). SBA is defined as childbirths assisted by qualified doctors, nurses/midwives/paramedics, family welfare visitors (FWVs), community skilled birth attendants (CSBAs), and medical assistants/sub-assistants’ community medical officers (MAs/SACMOs).

#### 2.2.2 Explanatory variables

Based on previous studies we selected the independent variables for this study were age at marriage in years, wealth index, administrative region, place of residence (rural/urban), age at first birth (measured in years), years of education, husband's or partner's years of education, number of antenatal care visit (no visit, 1–4 visit, more than 4 visit), the number of children ever born (birth order), birth interval in years, decision making power, and exposure to mass media [[39], [40], [41], [42], [43]]. Household wealth index consists of household's ownership of assets and dwelling characteristics such as televisions and bicycles; housing materials; and access to water and sanitation facilities. Using principal component analysis all assets were assigned a score, and finally, based on the continuous scale of assets score, all households were ranked into ten wealth quintiles [44]. On the basis of the existing literature, the variable ‘decision making power’ was categorised using the criteria whether a pregnant woman participated in decision-making related to (i) her own healthcare, (ii) making major household purchases and (iii) visits to her family and relatives [40,45,46]. The category ‘no decision’ meant that none of the three decisions were taken by the woman, ‘1–2 decisions’ indicated that a woman participated in making at least one or two decisions in the three mentioned areas and ‘all three decisions’ reﬂected that a woman participated in decision making in all the three areas.

We constructed an index of exposure to mass media using women's responses regarding the use of three forms of mass media (radio, television, and newspapers) in a typical week. For each medium, the frequency of media use was coded in the following ways: no use at all was coded 0, less than once a week was coded 1, and at least once a week was coded 2. We summed the scores for each medium and the score ranged from 0 to 6. Finally, the total score was divided into three groups as follows: 0 score means no exposure at all, 1–3 score categorised as irregular exposure, and 4–6 score represents regular exposure. Previous studies also used the similar index of exposure to mass media [29,42,46]. Although both the age at first marriage and marriage to first birth are significant, we used age at marriage in our decomposition analysis, because it is more sensible and robust in predicting delivery assisted by skilled birth attendants.

### 2.3 Statistical analysis

Firstly, a descriptive analysis devised to show the features of the potential determinants of increase in facility child births in Bangladesh, hereafter correlation between the dependant variables and explanatory variables as well as multicollinearity among the independent variables were tested. Results does not suggest any obvious multicollinearity (variance inflation factors<3 for all variables included in the model), variables are apposite to include into the regression model having VIFs less than 5 [47]. By applying chow test we checked the parameter stability across the five rounds, and hence we applied classical regression based decomposition analysis [48] instead of Oaxaca-Blinder (OB) decomposition [[49], [50], [51]] to show the contribution of different individual determinants in explaining the increase in SBA coverage for the pooled, rural, and urban samples respectively. As the outcome variable is binary, for assessing the relationships between skilled birth attendant (SBA) for mother i at time t (SBA_n,t_) let us define it as A, and the covariates (Wealth index, education, spouse education, number of ANC visits, birth order, age at marriage, mass media access, decision making power, prenatal doctor visit, current working status, birth interval, maternal BMI, years of marriage to first birth) say (X), logistic regression has been used, where location fixed effects is denoted by (μ_n_) as well as trend effects by (T). The coefficients (β) represents the parameters of interest. By adding a standard disturbance term ε_n,t_, we depicted the relationships by equation (1):

(1) $$A=Logit\left({SBA}_{n,t}\right)=\left(\ln\left({SBA}_{n,t}\right)\right)/\left(1-\ln\left({SBA}_{n,t}\right)\right)=\mu_{n}+\beta X_{n,t}+T+\varepsilon_{n,t}$$

The estimated parameters from equation (1) is now employed in equation (2) to do the decomposition analysis, where we only used variables which are significant at 5% level of significance. Since, the sample is clustered in nature standard errors were taken into consideration.

(2) $$\Delta A=\beta \;\left({\bar{X}}_{t=k}-{\bar{X}}_{t=1}\right)$$

For calculating equation (2), we set the 2004 BDHS round (t = 1) and the latest round 2017–18 (t = k). The decomposition results are obtained by multiplying mean or percentage observed changes of each explanatory variable over time by its regression coefficient. By doing such decomposition we get the predicted change in SBA from each change in a factor and thus presents individual variable contributions in explaining the changes in SBA. We presented our estimated logistic regression in Table 4. Pooling all five rounds together and using 2004 round as the base we accounted for trend effects through a series of year dummy variables. We also depicted separate logistic regressions for the sub-samples of rural and urban regions. We have used Stata 14.0 (Stata Corp, College Station, TX) for all data analyses. We applied svy command for sampling weight adjustment.

## 3 Results

In this study we analysed sample of 26,686 reproductive-aged women, 32.9% (8780) from the urban and 67.1% (17,906) from the rural area. In Table 1 we demonstrated the socio-demographic characteristics of women who gave birth of at least one live born child in the preceding three years of five rounds of BDHSs. The mean age at marriage for the pooled sample is 15.8 years. More women living in urban area had formal education than rural women (No formal education: 16.3% vs 24.0% respectively). Clear inequality in wealth quintile of rural and urban women (poorest: 30.7% vs 6.9%) and (richest: 5.4% vs 46.0%). There was also inequality in antenatal care, number of children, mass media exposure, decision making power, and spouse education among the urban and rural women.

**Table 1:** Socio-demographic characteristics of respondents for the full sample.

Variable	Pooled Sample	Rural sample	Urban sample
	%	%	%
Mean Age at marriage (SD)	15.8 (2.8)	15.6 (2.6)	16.5 (3.3)
Division			
Barisal	5.9	6.5	4.0
Chittagong	21.2	21.2	21.1
Dhaka	32.5	28.0	47.3
Khulna	9.8	10.1	8.8
Rajshahi	22.9	25.6	14.2
Sylhet	7.7	8.6	4.6
Women's years of education			
No formal education	22.2	24.0	16.3
1–5 years	30.3	31.2	27.2
6–12 years	44.8	43.3	49.7
More than 12 years	2.8	1.6	6.9
Mena years of women education (SD)	5.2 (4.6)	4.7 (4.4)	6.7 (4.9)
Wealth index (1–5)			
Poorest	25.1	30.7	6.9
Poorer	21.3	24.9	9.6
Middle	18.8	20.4	13.9
Richer	19.8	18.6	23.7
Richest	14.9	5.4	46.0
ANC visit number			
No ANC visit	30.5	34.8	16.5
1-4 visits	42.3	43.2	39.3
More than 4 visits	27.2	22.0	44.2
Birth order			
First	34.0	32.6	38.8
Second	28.9	28.1	31.7
Third	17.4	17.8	16.0
Fourth and above	19.7	21.5	13.5
Mass media access			
Not at all	35.1	41.3	14.9
Irregular	52.7	48.7	65.9
Regular	12.2	10.0	19.2
Decision making power			
No decision	25.4	27.0	20.3
1-3 decision	33.0	33.5	31.6
All 3 decision	41.6	39.6	48.1
Religion			
Muslims	91.7	91.7	91.6
Others	8.4	8.3	8.4
Prenatal doctor visit			
No	52.8	58.6	33.8
Yes	47.2	41.4	66.2
Mean years of husbands education (SD)	5.2 (4.6)	4.7 (4.4)	6.7 (4.9)

Note: Sampling weights has been considered in all estimations, SD=Standard deviation.

Table 2 presents trends of live births facilities among the women (aged 15–49). Delivery assisted by skilled birth attendants increased about 242.2% over the 14 years’ time span from 2004 to 2017–2018 in national level which were very fast but still under the expectation to reach the target of sustainable deployment goals (SDGs). The SDGs goal 3 target 3.1 aims to reduce MMR to less than 70 per 100,000 live births by 2030 [52]. The percentage change of facility child birth in rural areas were faster than urban areas, albeit slightly behind according to the point change up to 2014. From 2004 to 2017–2018 delivery assisted by SBAs in rural areas exceeded urban counterparts in case of point change. The tempo of increase was faster from 2007 onwards compared to the period 2004 to 2007.

**Table 2:** Changes in delivery assisted by skilled birth attendant for different samples across the five BDHS rounds.

Samples	Delivery assisted by skilled birth assistant		
	Total	Rural	Urban
2004	15.6	11.1	32.8
2007	20.6	15.3	40.3
2011	30.4	23.6	52.7
2014	44.0	37.1	63.8
2017	53.4	48.2	67.9
Change	37.8	37.1	35.1
% Change	242.2	334.9	107.2

Note: Sampling weights has been used in calculations.

Table 3 depicts that years of educational attainment improved for both men and women in Bangladesh, but it is nearly 1.5 times rapid for women than for man. During the 14 years’ time period birth interval increased 3.3 years from 2.9 years. Four plus antenatal care visits during the pregnancy has increased 3 times. Average age at first birth 2017–2018 BDHS stands 18.5 years and 17.4 years in 2004, which is not a mentionable change.

**Table 3:** Changes in the means or percentages of key variables from 2004 to 2017.

Year	2004	2017	Change	% Change
Wealth index (1–10)	3.8	6.1	2.3	60.5
Women's years of education	3.7	6.9	3.2	86.5
Husbands/partner years of education	4.1	6.3	2.2	53.7
ANC visit (more than 4)	16	47	31	193.8
Birth order/Parity	2.9	2.1	−0.8	−27.6
Age at marriage	15	16.4	1.4	9.3
Regular mass media access	25.2	4.9	−20.3	−80.6
Decision making power	26.1	53.4	27.3	104.6
Prenatal doctor visit	31.4	76	44.6	142.0
Marriage to first birth (years)	2.5	2.1	−0.4	−16.0
Current working status	18.5	37.4	18.9	102.2
Birth Interval in years	3.3	2.9	−0.4	−12.1
Maternal BMI	19.8	22.4	2.6	13.1

Note: Sampling weights has been used.

**Table 4:** Delivery assisted by skilled birth attendant's regressions pooled across years for various samples.

Model	Full sample (baseline)		Rural		Urban	
	Coeff.	p-value	Coeff.	p-value	Coeff.	p-value
Wealth index	0.107	0.000	0.076	0.000	0.073	0.000
Women's education (yrs)	0.068	0.000	0.065	0.000	0.093	0.000
Husbands education (yrs)	0.036	0.000	0.032	0.000	0.053	0.000
ANC visit (more than 4)	0.587	0.000	0.536	0.000	0.639	0.000
Birth order	−0.166	0.000	−0.201	0.000	−0.056	0.074
Age at Marriage	0.064	0.000	0.063	0.000	0.064	0.000
Mass media access	0.135	0.000	0.103	0.003	0.246	0.000
Decision making power	0.001	0.966	0.025	0.305	−0.108	0.010
Prenatal doctor visit	0.712	0.000	0.760	0.000	0.578	0.000
Current working status	−0.198	0.000	−0.141	0.003	−0.382	0.000
Birth intervals (years)	0.013	0.034	0.008	0.263	0.018	0.119
Maternal BMI	0.061	0.000	0.057	0.000	0.059	0.000
Marriage to first birth (yrs)	0.063	0.000	0.068	0.000	0.057	0.001
Year 2007	0.094	0.136	0.094	0.214	0.208	0.072
Year 2011	0.528	0.000	0.557	0.000	0.660	0.000
Year 2014	0.912	0.000	0.958	0.000	1.014	0.000
Year 2017	0.917	0.000	0.990	0.000	1.042	0.000
Pseudo R-squared	0.292		0.256		0.293	
Area Under ROC	0.845		0.830		0.844	
N	26,686		17,906		8780	

We find moderately large impacts of household wealth on delivery assisted by skilled birth attendants with the impact of a 1-point increase in the wealth index (measured on a 1–10 scale) usually predict the increase of log odds of delivery assisted by skilled birth attendants by 0.1 point (Table 4). In other words, the increase of delivery assisted by skilled birth attendants differ between a pregnant woman in the poorest household in our sample and the richest is about log odds of 1.0 standard deviations. For one years of increase in women education, the log odds of being a delivery assisted by skilled birth attendants increased by around 0.07 for the pooled sample. Husbands or partner's education also has significant effects on delivery assisted by SBAs, as it is increased by about a factor of log odds of 0.04 for each year additional attainment of academic calendar. Increase of years of education both women and their spouse, antenatal care visits, household wealth, age at first birth, antenatal care visits are positively associated with SBA for the whole and sub-samples. There is no significant association between women empowerment variable decision making power and increase in utilising the facility of skilled birth attendants. All the regression models presented in Table 4 and table A1 (appendix) showed area under the receiver operating characteristic curve (AUROC) above 0.80, i.e. has more than 80% overall accuracy to correctly classify delivery assisted by SBA vs without SBA.

In table A1 (presented in appendix) we checked coefficients stability across the waves. In the last column we presented the results of so called chow test used as an indication of coefficients stability. Since there was minimum variation in certain variables between the beginning and the end rounds, we applied classical decomposition instead of Oaxaca-Blinder (OB) decomposition. Table 5 reveals three interesting and important findings. Firstly, our pooled model explains about four-fifths (78.02%) of the actual change in SBA at delivery, so we can deduce that this model is fairly robust. Secondly, wealth index and education explain about 38.2% of the predicted change. Thirdly health indicators (e.g., more than four ANC visits and prenatal doctor visits) aggregately explaining over one-third (35.1%) of the total predicted change. Birth order and age at marriage jointly explains about 17.4% of the predicted change in SBA at delivery.

**Table 5:** Decomposing sources of skilled birth attendants at delivery change for the full sample, 2004 to 2017–2018.

Year	Estimated coefficient (1)	Sample Means: 2004	Sample Means: 2017	Change in means (2)	Predicted Change (1) × (2)	Share of predicted Change (%)
SBA (Outcome)		−1.688	0.136	1.824	1.423	
Wealth index (1–10)	0.107	3.8	6.1	2.3	0.25	17.3
Women's education (yrs)	0.068	3.7	6.9	3.2	0.22	15.3
Spouse education (yrs)	0.036	4.1	6.3	2.2	0.08	5.6
ANC visit (more than 4)	0.587	16	47	31.0	0.18	12.8
Birth order/Parity	−0.166	2.9	2.1	−0.8	0.13	9.3
Age at marriage	0.064	15	16.4	1.4	0.09	6.3
Prenatal doctor visit	0.712	31.4	76	44.6	0.32	22.3
Maternal BMI	0.061	19.8	22.4	2.6	0.16	11.1
Ratio of predicted SBA change to actual					78.02%	100

Note: We obtained sample mean in 2004 and 2017–2018 as taking the Ln of ratio of the proportions of delivery assisted by skilled birth attendants and the proportions of delivery without the assistance of skilled birth attendants. Questions may be arising why we did this mathematics? We used logistic regression coefficients to estimate the predicted change in utilising SBAs at delivery (where we utilised linear relation in the right hand side of the logistic regression equation). So, we apply the mathematical principles to apply same linearization in both sides.

**Fig. 2:**
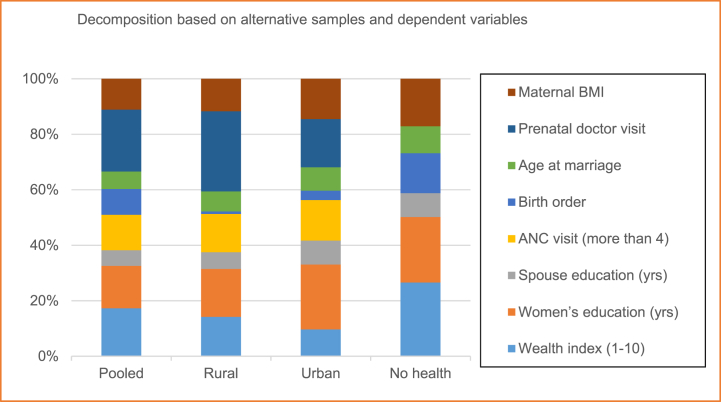
Contribution of factors in explaining the increase of SBAs at delivery.

**Fig. 3:**
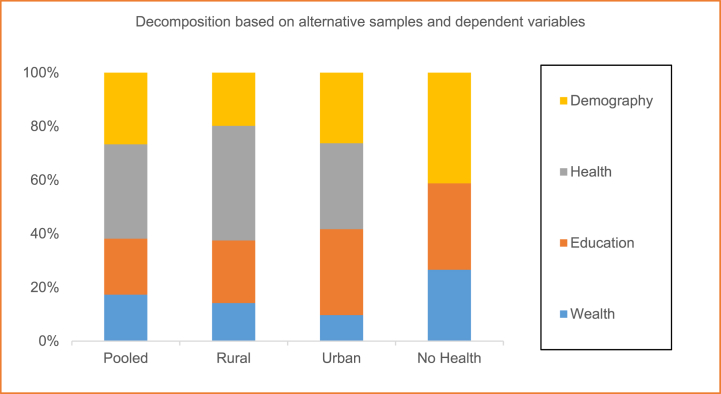
Contribution of aggregated factors in explaining the increase of SBAs at delivery.

In Fig. 3 we reported aggregated effects (e.g., wealth index is termed as “wealth”; women's years of education and husbands or partner's years of education are aggregated as “education”; more than four number of antenatal care visits and prenatal doctor visits are combined as “health”; birth order, age at marriage and maternal BMI grouped as “Demography”). We also documented the individual contributions of each determinant to the total predicted change (In Fig. 2 and table A3). In case of the rural sample we observe more or less same amount of contribution of all the explanatory variables without two exceptions. Firstly, the contribution of birth order is only 0.09%. However, the contribution of prenatal doctor visit is more than one-fourth (28.9%). Secondly, the predictive power of the model is approximately 61.33% which is the lowest compared to the baseline full sample and the urban sample. But switching to the urban model, we perceive women's education contributing the highest amount of 23.4% as well as aggregately “education” explaining one-third (33.1%) of the change. Finally, if we exclude health variables from the model on the ground that it has not changed up to the expected level, then demography, education and wealth explain 41.2%, 32.2% and 26.6% respectively.

## 4 Discussion

Our study demonstrated an increasing trend of skilled attendance at birth in Bangladesh between 2004 and 2017–18. Most of This improvement in SBA (78.0% explained) was resulted by improvements in socio-demographic factors, wealth status, health and education. Our study showed that, women education improved remarkably. This development reflects the results of various stipend program initiatives taken by the government of Bangladesh to promote education among the women, by doing so the authorities are trying to improve child health and maternal health in the long run. It has already proved that the educational improvement is contributing largely to achieve SDG targets in health sectors. Because educated women are more conscious about their maternal health and child nutrition than the uneducated counterpart. In our study we found improvement of skilled birth attendant at delivery as a results of improvement of antenatal care visits and prenatal doctor visits along with the improvement of women education and socioeconomic status. It is possible to improve antenatal care visit and prenatal doctor visit as much as we wish according to our health sector development, and by doing so we can increase the percentage of skilled birth attendant at birth.

Our study also depicted that, the interval between births has increased, which refers the success of family planning program of Bangladesh government. It implying that the mothers are becoming more concern about their existing children health as well as their own health. Birth order has decreased and age at marriage has increased, which also representing another successful contribution of family planning program. However, the tempo of increase in age at marriage is not up to the expected level, which is considered as an influential determinant to reduce both child and maternal mortality rate.

An interesting finding is that the regular mass media access has decreased largely over the time span. This may be due to the reason that the BDHS considered the frequency of reading newspaper, watching television, and listening radio to measures the mass media access, but for the last few years the use of smart phone, tablet, iPhone etc. has boomed in Bangladesh. So, the tendency of reading printed newspaper, watching television, and hearing radio has rapidly declined among the people as well as pregnant women, instead they are reading online newspapers, browsing various social medias, and watching movies and dramas by using smart phone. Sizeable increase has observed in the frequency of both the factors of prenatal doctor visits and decision making power, and it is undoubtedly promoting delivery assisted by skilled birth attendants. No mentionable improvement in age at marriage, clearly indicating existence of early marriage in Bangladesh and special program should be taken to eradicate it.

Our decomposition analysis for the full sample model as well as different sub-samples showed that education, wealth, health and demographic factors are the largest drivers in explaining increase in facility child birth in Bangladesh. Some other studies also found the same results in increase of facility child birth and determined that wealth and education is mainly responsible for the existing inequalities [31,33,53]. So, for the expected and sustainable improvement in facility child birth the authority should give importance in development of these determinants. Our study reveals that birth order is an important determinant in improved use of facility child birth which is in congruent of some other previous studies [[54], [55], [56]]. Maternal body mass index is also appeared as a dominant player in utilization of skilled birth attendants during the time period. The story behind this scenario maybe due to the increased prevalence of overweight and obesity as a results of less physical activities and habituation of westernized lifestyles during the last few years.

The decomposition analysis for different sub-samples of urban, rural and omitting the health variable shows factor have varying contribution in explaining the increased use of skilled birth attendants between 2004 and 2017–18. Pervious literature also demonstrated that there was inequality in access of skilled birth attendants by place of residence [[55], [57], [58], [59], [60], [61], [62]]. In the urban region increased in skilled birth utilization is highly influenced by education for both women and their spouses, birth order, age at marriage and maternal BMI. On the other hand, wealth and prenatal doctor visits are more influentially contributing in the rural Bangladesh. The reason maybe for the last two decades the socioeconomic condition of the countryside peoples and community medical facilities have improved. Therefore, based on the regression based decomposition results it is evident that improvement in wealth, education, health facilities (number of antenatal care visits, prenatal doctor visits), number of children born, increased age of marriage, apposite maternal BMI should be in supreme priority in policy making for achieving the SDGs target within 2030 in utilization of skilled birth attendants in delivery.

## 5 Strengths and limitations of the study

This study consists of some strengths as well as some drawbacks. The first strength is that, we analysed a nationally representative large cross-sectional data sets from the reliable source of BDHS. Secondly, we used classical decomposition to determine the contribution of each factors in explaining the contribution of increase of SBA at delivery which would help policymakers for implementing the appropriate interventions. The first limitation is that, we failed to include some established factors e.g. complications related to delivery distance to the nearest hospital, transportation issues to reach health facilities, presence of SBAs in the geographic area, and cost of SBAs due to the unavailability in BDHS. Secondly, since the data are cross-sectional and collected retrospectively the causality may not be strongly established. Thirdly, we used the socioeconomic status of women by wealth index, which is a proxy and constructed based on household construction materials, household utilities, arable land, livestock's, and other amenities. So, it may overestimate or underestimate the actual socioeconomic status.

## 6 Conclusion

Skilled birth attendant at delivery has improved for the last two decades in Bangladesh. There are available scopes to facilitate this improvement with more velocity by identifying and improving some determinants to reach the recommended level of 50% and to fulfill the SDG targets. In this study we identified the factors influencing the improvement in skilled birth attendants at delivery and their individual contribution. We found that, education, household wealth, antenatal care visit, prenatal doctor visit and some demographic factors are the vital drivers for increased in skilled birth attendant at delivery. Two health determinants: antenatal care visit and prenatal doctor visit (skilled doctor not skilled medical care provider) are the extensive drivers, which is modifiable and can contribute rapidly in the improvement of attendant of skilled personnel at childbirths. So, program should be initiate and interventions must be address to immediate development of these two determinants to accelerate skilled birth attendant at delivery up to the desired target.

## Declarations

### Author contribution statement

Md Sohel Rana: Conceived and designed the experiments; Performed the experiments; Analysed and interpreted the data; Contributed reagents, materials, analysis tools or data; Wrote the paper.Sk. Masum Billah: Conceived and designed the experiments.Mohammed Moinuddin: Md. Abu Bakkar Siddique: Contributed reagents, materials, analysis tools or data.Md. Mobarak Hossain Khan: Wrote the paper.

### Data availability statement

The authors do not have permission to share data.

### Additional information

No additional information is available for this paper.

## Declaration of competing interest

The authors declare that they have no known competing financial interests or personal relationships that could have appeared to influence the work reported in this paper.

## Figures and Tables

**Table A1:** Socio-demographic characteristics of respondents for the full sample

Variable	2004		2007		2011		2014		2017	
	Rural	Urban	Rural	Urban	Rural	Urban	Rural	Urban	Rural	Urban
	%	%	%	%	%	%	%	%	%	%
Mean Age at marriage (SD)	14.8 (2.4)	15.5 (3.0)	15.2 (2.5)	16.3 (3.2)	15.6(2.5)	16.5 (3.4)	16.1 (2.6)	17.0 (3.6)	16.2 (2.5)	17.0 (3.2)
Division										
Barisal	6.8	3.6	7.0	4.1	6.4	3.5	5.9	5.6	6.5	3.5
Chittagong	19.8	22.7	20.7	22.0	21.8	20.7	21.6	22.5	21.9	18.5
Dhaka	27.7	43.8	28.0	45.6	26.6	47.9	31.5	46.2	27.4	51.7
Khulna	11.5	10.6	10.5	9.4	10.1	8.9	8.3	7.4	9.6	8.1
Rajshahi	26.4	13.9	25.0	14.9	27.4	15.1	22.2	12.8	25.6	13.9
Sylhet	7.8	5.4	8.8	4.0	7.8	3.9	10.5	5.5	8.9	4.3
Women's years of education										
No formal education	41.1	30.8	30.9	21.4	22.0	13.6	15.3	11.0	7.5	8.3
1–5 years	32.4	30.0	28.6	26.7	32.6	27.3	31.0	24.9	30.7	27.0
6–12 years	26.1	36.0	37.5	43.3	44.4	53.1	52.2	56.3	59.2	56.0
More than 12 years	0.5	3.3	3.0	8.6	1.0	6.1	1.5	7.8	2.5	8.7
Women's education mean (SD)	3.4 (3.5)	4.7 (4.2)	4.5 (4.0)	6.1 (4.6)	5.1 (3.6)	6.7 (4.2)	5.8 (3.6)	7.1 (4.1)	6.7 (3.4)	7.4 (4.2)
Wealth index (1–5)										
Poorest	48.2	13.9	37.5	10.3	28.7	5.0	24.8	3.9	11.9	3.6
Poorer	27.4	16.6	24.9	10.6	24.9	7.8	23.2	7.3	24.0	6.9
Middle	15.7	14.3	17.7	16.0	22.4	14.1	20.8	14.7	25.1	10.6
Richer	6.4	19.9	16.2	22.3	19.5	24.4	23.3	24.2	29.6	26.6
Richest	2.3	35.3	3.8	40.8	4.5	48.6	8.0	49.9	9.5	52.3
ANC visit number										
No ANC visit	49.0	25.0	43.8	24.2	40.4	19.0	25.5	10.5	9.0	5.3
1-4 visits	39.8	41.0	40.3	37.6	41.7	37.9	48.3	44.2	48.0	36.6
More than 4 visits	11.2	34.0	15.9	38.2	17.8	43.1	26.2	45.3	43.1	58.2
Birth order										
First	26.7	30.8	31.6	33.5	31.5	40.3	37.8	45.8	37.1	41.3
Second	25.0	27.4	25.6	31.0	28.7	32.5	29.5	31.7	32.2	35.0
Third	18.2	19.4	16.9	17.3	18.8	16.0	17.2	13.6	17.5	14.4
Fourth and above	30.1	22.5	25.9	18.2	21.0	11.2	15.6	8.9	13.3	9.4
Mass media access										
Not at all	36.3	13.6	42.0	17.0	42.2	12.5	47.2	12.8	39.7	19.5
Irregular	42.2	46.8	43.7	58.6	52.0	72.8	48.1	73.7	56.9	71.7
Regular	21.5	39.6	14.3	24.4	5.8	14.7	4.7	13.5	3.5	8.8
Decision making power										
No decision	37.9	29.2	21.0	15.4	28.3	20.3	28.6	22.6	17.0	14.5
1-3 decision	37.6	39.0	36.5	36.9	32.4	28.7	28.8	29.0	31.4	27.2
All 3 decision	24.6	31.8	42.6	47.7	39.3	51.0	42.6	48.5	51.6	58.3
Religion										
Muslims	91.8	92.2	91.2	92.1	91.4	91.6	92.6	89.3	91.4	92.9
Others	8.2	7.8	8.8	7.9	8.6	8.4	7.4	10.8	8.6	7.1
Prenatal doctor visit										
No	74.3	46.7	69.9	43.9	65.9	39.6	48.0	25.9	27.5	14.7
Yes	25.7	53.3	30.1	56.1	34.1	60.4	52.0	74.1	72.5	85.3
Husband's education Mean (SD)	3.7 (4.1)	5.6 (4.9)	4.3 (4.6)	6.3 (5.1)	4.7 (4.4)	6.9 (4.9)	5.1 (4.3)	7.2 (4.8)	6.0 (4.4)	7.3 (4.9)

Note: Sampling weights has been considered in all estimations, SD=Standard deviation.

**Table A2:** Delivery assisted by skilled birth attendant's (SBA) regressions for the full sample by round with tests for coefficient differences over time

Model	2004		2007		2011		2014		2017		Sig. Diff.?
	Coeff.	p-value	Coeff.	p-value	Coeff.	p-value	Coeff.	p-value	Coeff.	p-value	
Wealth index (1–10)	0.155	0.000	0.126	0.000	0.134	0.000	0.108	0.000	0.089	0.000	No
Women's years of education	0.088	0.000	0.066	0.000	0.059	0.000	0.066	0.000	0.086	0.000	No
Husbands years of education	0.032	0.019	0.041	0.001	0.039	0.000	0.049	0.000	0.036	0.000	No
ANC visit (more than 4)	0.676	0.000	0.608	0.000	0.657	0.000	0.522	0.000	0.558	0.000	No
Birth order	−0.047	0.179	−0.170	0.000	−0.170	0.000	−0.212	0.000	−0.147	0.000	No
Age at marriage	0.070	0.000	0.077	0.000	0.084	0.000	0.072	0.000	0.042	0.003	No
Mass media access	0.048	0.498	0.165	0.020	0.157	0.010	0.097	0.182	0.343	0.000	Yes
Decision making power	−0.049	0.405	0.031	0.601	−0.029	0.451	0.056	0.188	0.057	0.211	Yes
ANC visit (at least 1)	0.554	0.000	1.085	0.000	0.482	0.000	0.627	0.000	1.135	0.000	No
Current working status	−0.213	0.095	−0.151	0.154	−0.221	0.030	−0.254	0.003	−0.222	0.002	Yes
Birth intervals (years)	−0.010	0.602	0.034	0.043	0.033	0.006	0.041	0.001	0.015	0.188	No
Marriage to first birth (years)	0.081	0.000	0.085	0.000	0.073	0.000	0.076	0.000	0.044	0.013	Yes
Pseudo R-squared	0.263		0.309		0.234		0.210		0.207		
Area under ROC	0.834		0.856		0.810		0.793		0.795		
N	5273		4865		7143		4444		4920		

Note: In all estimation we used sampling weights.

**Table A3:** Decomposition based on alternative samples and dependent variables

Dependent variable	SBA	SBA	SBA	SBA
Area	All	Rural	Urban	All
Model	Full	Full	Full	No health
Wealth index (1–10)	17.3%	14.2%	9.7%	26.6%
Women's years of education	15.3%	17.3%	23.4%	23.6%
Spouse years of education	5.6%	6.0%	8.6%	8.6%
ANC visit (more than 4)	12.8%	13.8%	14.6%	
Birth order	9.3%	0.9%	3.4%	14.4%
Age at marriage	6.3%	7.2%	8.4%	9.7%
Prenatal doctor visit	22.3%	28.9%	17.4%	
Maternal BMI	11.1%	11.7%	14.5%	17.1%
Sum	100%	100%	100%	100%
Aggregated sources of predicted change				
Wealth	17.3%	14.2%	9.7%	26.6%
Education	20.9%	23.3%	32.0%	32.2%
Health	35.1%	42.7%	32.0%	
Demography	26.7%	19.8%	26.3%	41.2%
Sum	100%	100%	100%	100%
Predictive power of model				
(1) Actual change in SBAs at delivery	1.824	2.008	1.466	1.45
(2) Predicted change in SBAs at delivery	1.423	1.232	1.059	0.88
Predictive power (%): (2)/(1)	78.02%	61.33%	72.22%	60.8%
